# Ultra‐High Temperature Treatment and Storage of Infant Formula Induces Dietary Protein Modifications, Gut Dysfunction, and Inflammation in Preterm Pigs

**DOI:** 10.1002/mnfr.202200132

**Published:** 2022-09-12

**Authors:** Jing Sun, Halise Gül Akıllıoğlu, Karoline Aasmul‐Olsen, Yuhui Ye, Pernille Lund, Xiao Zhao, Anders Brunse, Christian Fiil Nielsen, Dereck E. W. Chatterton, Per Torp Sangild, Marianne N. Lund, Stine Brandt Bering

**Affiliations:** ^1^ Comparative Pediatrics and Nutrition Department of Veterinary and Animal Sciences University of Copenhagen Dyrlægevej 68 Frederiksberg C 1870 Denmark; ^2^ Department of Food Science University of Copenhagen Rolighedsvej 26 Frederiksberg 1958 Denmark; ^3^ Arla Foods Ingredients Sønderhøj 10–12 Viby J 8260 Denmark; ^4^ Department of Pediatrics and Adolescent Medicine Rigshospitalet Blegdamsvej 9 Copenhagen Ø 2100 Denmark; ^5^ Hans Christian Andersen Children's Hospital J. B. Winsløws Vej 4 Odense C 5000 Denmark; ^6^ Department of Biomedical Sciences University of Copenhagen Blegdamsvej 3B Copenhagen N 2200 Denmark

**Keywords:** gut inflammation, liquid infant formula, Maillard reaction, preterm infants, protein modification

## Abstract

**Scope:**

Ready‐to‐feed liquid infant formula is increasingly used for preterm infants when human milk is unavailable. These formulas are sterilized by ultra‐high temperature treatment, but heating and storage may reduce bioactivity and increase formation of Maillard reaction products with potential negative consequences for immature newborns.

**Methods and results:**

Using preterm pigs as a model for sensitive newborn infants, the study tests the intestinal responses of feeding experimental liquid formula within 5 days. A pasteurized formula (PAST) with the same nutrient composition but less protein modifications serves as control to ultra‐high temperature‐treated formula without (UHT) and with prolonged storage (SUHT). Relative to PAST, UHT contains lower levels of lactoferrin and IgG. Additional storage (40 °C, 60 days, SUHT) reduces antimicrobial capacity and increases non‐reducible protein aggregates and Maillard reaction products (up to 13‐fold). Pigs fed SUHT have more diarrhea and show signs of intestinal inflammation (necrotizing enterocolitis) compared with pigs fed PAST and UHT. These clinical effects are accompanied by accumulation of Maillard reaction products, protein cross‐links, and inflammatory responses in the gut.

**Conclusion:**

The results demonstrate that feeding UHT infant formulas, particularly after prolonged storage, adversely affects gut maturation and function in preterm pigs used as a model of preterm infants.

## Graphical abstract

Ready‐to‐feed infant formulas are often used for preterm infants when human milk is unavailable. These formulas are treated with ultra‐high temperature, but during storage, protein modifications are induced. The study shows that stored formulas adversely affect gut maturation and function in preterm pigs. The clinical consequences of feeding infant formulas with a high degree of protein modifications for preterm infants are unknown.

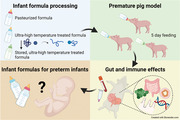

## 1 Introduction

Infant formula (IF) feeding is needed to feed preterm infants when human milk is not available. Although mother's own milk or alternatively donor human milk is the optimal strategy for feeding newborn preterm infants,^[^ ^1^ ^]^ mother's milk is often in short supply after preterm birth and human milk banks are not available in all neonatal intensive care units worldwide. Powdered IF is most often used, but due to concern about risk of transmission of pathogens from contamination, the use of ready‐to‐feed liquid IFs is gradually increasing in the neonatal intensive care units.^[^ ^2^, ^3^ ^]^ Ready‐to‐feed liquid IFs are sterilized by ultra‐high temperature (UHT) treatment to eliminate pathogens and extend shelf‐life. The extensive UHT treatment induces undesirable protein aggregation and chemical modifications caused by Maillard reaction,^[^ ^4^, ^5^ ^]^ leading to the formation of Maillard reaction products (MRPs), including Amadori compounds, α‐dicarbonyl compounds and advanced glycation end products (AGEs).^[^ ^6^, ^7^ ^]^ The Maillard reaction is accelerated with increased temperature and time of storage.^[^ ^6^, ^8^, ^9^ ^]^ This is a challenge especially for regions with a hot climate and for overseas transportation where temperatures can often reach up to 60 °C for months.^[^ ^10^ ^]^

In adults, AGEs have been linked to several disorders including diabetes, obesity, atherosclerosis, chronic kidney disease, intestinal bowel disease, and Alzheimer's disease.^[^ ^11^, ^12^ ^]^ Due to insufficient chemical characterization of AGEs, it is unknown how dietary AGEs, relative to physiologically formed AGEs, contribute to these pathophysiological disorders.^[^ ^13^ ^]^ Skim milk powder with higher carboxymethyl lysine (CML) content has been shown to reduce body weight and increase inflammatory responses in rats,^[^ ^14^ ^]^ and chronic exposure to dietary CML in mice have led to deposition of CML in several organs.^[^ ^15^ ^]^ Moreover, dietary AGEs are metabolized by the colonic microbiota^[^ ^16^, ^17^, ^18^ ^]^ and damage the colon epithelial barrier in rats, which may enable excreted endotoxins to enter the systemic circulation.^[^ ^19^ ^]^

The level of MRPs in IFs are many‐fold higher than in breast milk, and preterm infants that receive IF are known to have a higher risk of feeding intolerance, gut dysfunctions, and necrotizing enterocolitis (NEC) than those receiving breast milk.^[^ ^20^ ^]^ Preterm infants are likely more sensitive to MRPs considering their immature gut and immune system, but it remains unclear if MRPs contribute to the unfavorable effects of IFs.^[^ ^21^, ^22^ ^]^ We have previously shown that a reduced heat load of whey protein ingredients for IF retained native proteins and improved gut maturation in preterm pigs.^[^ ^23^, ^24^ ^]^ However, the biological effect of UHT‐treated IF in sensitive newborns is still unknown.

Pigs born at 90% gestational age display an immature intestine and immune system similar to preterm infants.^[^ ^25^ ^]^ They spontaneously develop gut dysfunction and NEC and are highly sensitive to dietary interventions,^[^ ^24^, ^26^ ^]^ which makes them an appropriate model to investigate the physiological impact of UHT‐treated IF in preterm newborns. We hypothesized that IF subjected to indirect UHT treatment and storage will lead to unfavorable gastrointestinal effects in preterm newborn pigs, used as a model for infants. Ready‐to‐feed liquid IFs that are increasingly used to feed the very sensitive preterm infants when mother's milk is unavailable may have a considerable content of MRPs and other protein modifications.^[^ ^7^ ^]^ The physiological consequences of feeding very sensitive preterm infants an IF with high levels of MRPs are currently unknown. Considering the immature gut, these components may contribute to the adverse effects of IF feeding. This is the first pre‐clinical study to show that feeding UHT‐treated and stored IFs with increased levels of MRPs and protein modifications to premature piglets, a relevant pre‐clinical model, leads to increased inflammation and accumulation of AGEs in the gut.

## 2 Results

### 2.1 Changes in Protein Structure and Bioactivity in IF

**Figure 1:**
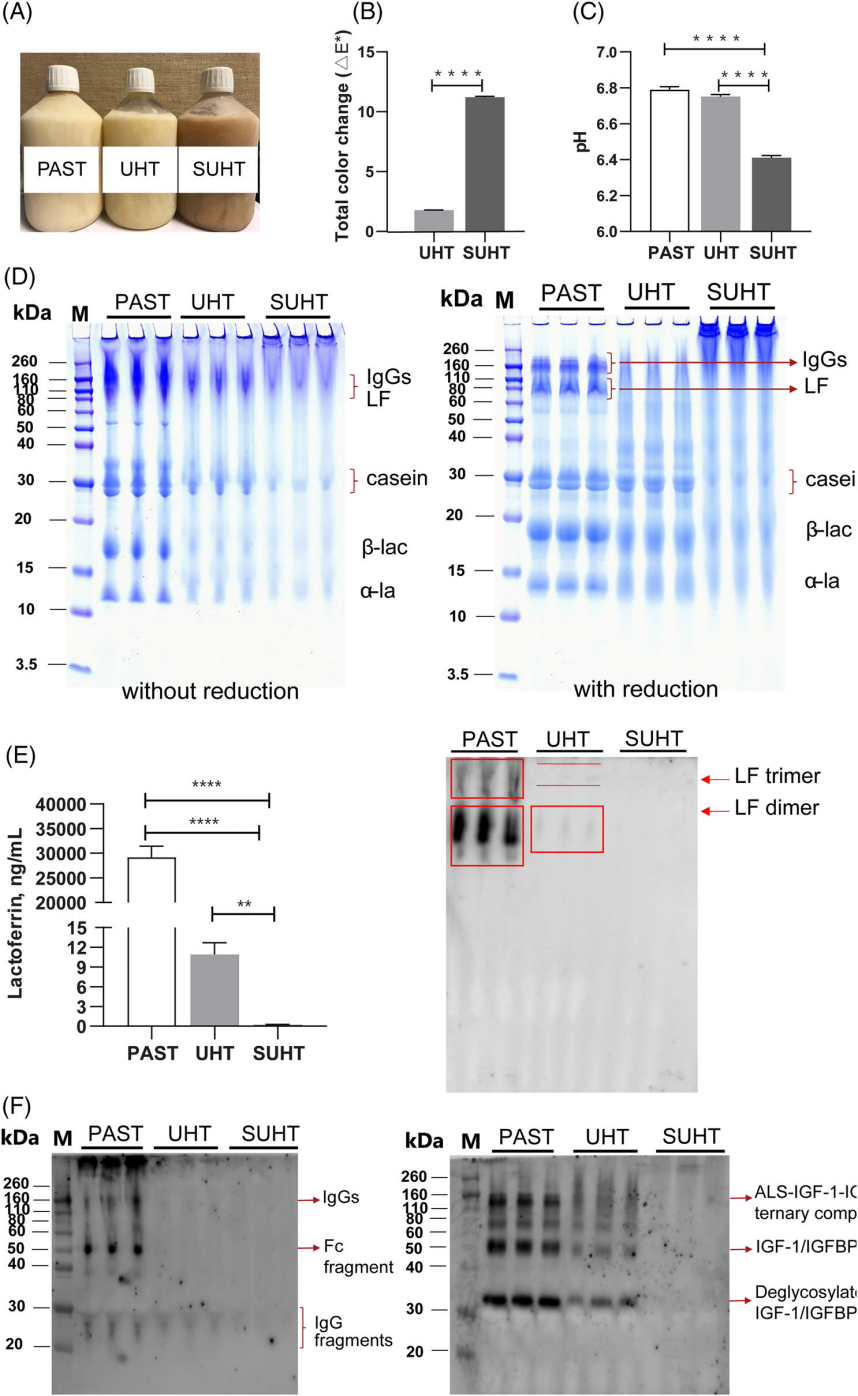
Characterization of the experimental infant formulas (IFs). Representative images of the bottled frozen IFs A), total color change relative to PAST IF^[^ ^4^ ^]^ B), pH of experimental IFs^[^ ^4^ ^]^ C), SDS‐PAGE with and without reduction^[^ ^5^ ^]^ D), lactoferrin^[^ ^5^ ^]^ (LF) concentration in experimental IFs determined by ELISA and western blot^[^ ^5^ ^]^ E), and semi‐quantification of IgG and insulin‐like growth factor (IGF)‐1 under non‐reducing and reducing conditions, respectively, using western blot^[^ ^5^ ^]^ F). Values are mean ± SE. *p < 0.05; ****p < 0.0001. α‐la, α‐lactalbumin; β‐lac, β‐lactoglobulin; ALS, acid‐labile subunit; BP, binding protein; PAST, pasteurized liquid infant formula (72 °C, 10 s); SUHT, UHT‐treated formula stored at 40 °C for 60 days; UHT, indirect UHT‐treated liquid infant formula (143 °C, 6 s).

Pasteurized IF (PAST) had a color similar to that of regular milk, whereas the total color change (ΔE*) had increased for ultra‐high temperature‐treated IF (UHT) and was further increased after storage at 40 °C for 60 days (SUHT, *p* < 0.0001), indicating formation of MRPs (**Figure** **1**A,B). The pH values were similar between PAST and UHT, but was decreased in SUHT (*p* < 0.0001, Figure 1C).

Formation of disulfide‐ and non‐reducible crosslinks of proteins was evaluated by SDS‐PAGE (Figure 1D). Under non‐reduced conditions, UHT had a loss of non‐aggregated proteins indicated by slightly lower number of protein bands and lower intensity (e.g., casein, β‐lactoglobulin, α‐lactalbumin) compared to PAST. In SUHT, most of the proteins were involved in aggregate formation and only faint protein bands were observed on the gel. After reduction of disulfide cross‐links under reducing conditions, the abundance of high molecular weight smears (>70 kDa) in PAST and UHT was largely decreased, and only a few faint smears remained in UHT. This indicates that most aggregates were disulfide‐linked (reducible) and that UHT treatment appeared to cause formation of some non‐reducible aggregates as the protein bands in UHT were more smeared than those in PAST. In contrast, a large amount of non‐reducible high molecular weight aggregates was observed in SUHT. Together, these data indicate a substantial loss of native protein and the formation of large and complex non‐reducible aggregates in SUHT compared to PAST.

The abundance of bioactive proteins was markedly affected by UHT treatment. Under both reducing and non‐reducing conditions, IgGs (≈160 kDa) and lactoferrin (LF, ≈80 kDa) were clearly visible in PAST and these protein bands could hardly be identified in UHT (Figure 1D). The loss of IgGs and LF after UHT treatment was confirmed by Western blot analysis and quantitative ELISA (Figure 1E,F). For PAST, most IgG was included in the high molecular weight aggregates at the top of the gel, but free IgG (≈160 kDa), the Fc part of IgG (≈50 kDa), and small fragments (20–30 kDa) were also visualized. However, only the small fragments were detectable in UHT and SUHT, and immunoreactivity was eliminated in SUHT. Immunoreactivity of pro IGF‐1 was not detectable in any of the samples under non‐reducing conditions, and was only detected and partly retained in UHT under reducing conditions.

### 2.2 Formation of MRPs and Amino Acid Cross‐Links in IF

UHT treatment caused a 2–8 fold increase in α‐dicarbonyl compounds, the intermediate products formed during the Maillard reaction and carbohydrate degradation (**Table** **1**). Concentrations of furosine, which is a marker for the Amadori product (early MRP), and AGE (except carboxyethyl lysine, CEL) were up to 6.5 times higher in UHT than PAST. The storage further increased furosine and AGE concentrations, with the highest increases observed for CML and methylglyoxal derived hydroimidazolone (MG‐H) whereas the α‐dicarbonyl compounds were decreased and diacetyl only detected in UHT. Pentosidine, methylglyoxal lysine dimer (MOLD) and glyoxal lysine dimer (GOLD) were not detected in any of the samples. Concentrations of lysinoalanine (LAL) and lanthionine (LAN) were only marginally increased in UHT compared to PAST, but the levels markedly increased in SUHT (2.4 and 5 times, respectively). Available lysine and arginine concentrations decreased by 10% in SUHT samples compared to PAST. Altogether, the results show that UHT treatment and particularly following storage led to structural and chemical modifications of proteins, namely aggregate formation, amino acid cross‐links, and MRP formation.

**Table 1:** Products derived from heat induced‐protein modification in the experimental infant formulas, PAST, UHT, and SUHT.^[^ ^7^ ^]^

	PAST	UHT	SUHT
Maillard reaction products			
Furosine [µg mL^–1^] (µg g^–1^ protein)	27 ± 0.9 (1797 ± 59)	42 ± 2.0 (2798 ± 136)	66.2 ± 2.8 (4413 ± 186)
CML [ng mL^–1^] (µg g^–1^ protein)	563 ± 30 (38 ± 2)	3635 ± 136 (242 ± 9)	7523 ± 493 (502 ± 33)
CEL [ng mL^–1^] (µg g^–1^ protein)	1069 ± 166 (71 ± 11)	1070 ± 22 (71 ± 1)	2039 ± 229 (136 ± 15)
MG‐H3 eqv. [ng mL^–1^] (µg g^–1^ protein)	962 ± 29 (64 ± 2)	1693 ± 47 (113 ± 3)	3179 ± 44 (212 ± 3)
GO‐H1 eqv. [ng mL^–1^] (µg g^–1^ protein)	ND	160 ± 40 (11 ± 3)	636 ± 80 (42 ± 5)
α‐Dicarbonyls			
Glyoxal [ng mL^–1^ sample]	176.8 ± 0.4	573.9 ± 39.9	49.1 ± 10.8
Methylglyoxal [ng mL^–1^ sample]	111.5 ± 14.3	333.4 ± 51.3	51.1 ± 5.3
Diacetyl [ng mL^–1^ sample]	ND	10.6 ± 0.7	ND
3‐DG [ng mL^–1^ sample]	985 ± 46	5509 ± 20	3762 ± 132
3‐DGal [ng mL^–1^ sample]	509 ± 4	4388 ± 2	1556 ± 13
Glucosone [ng mL^–1^ sample]	425 ± 2	802 ± 8	361 ± 1
Galactosone [ng mL^–1^ sample]	186 ± 7	368 ± 19	45 ± 3
AA derived cross‐links			
LAL [ng mL^–1^] (µg g^–1^ protein)	3911 ± 1214 (261 ± 81)	2974 ± 28 (198 ± 2)	9385 ± 269 (626 ± 18)
LAN [ng mL^–1^] (µg g^–1^ protein)	192 ± 88 (13 ± 6)	357 ± 41 (24 ± 3)	959 ± 134 (64 ± 9)
Available amino acids			
Lys [g 100 g^−1^ protein]	9.1 ± 0.5	9.3 ± 0.3	8.2 ± 0.1
Arg [g 100 g^–1^ protein]	2.1 ± 0.3	2.2 ± 0.0	2.0 ± 0.1

Results are presented as mean ± SD of two separate measurements. 3‐DG, 3‐deoxyglucosone; 3‐DGal, 3‐deoxygalactosone; AA, amino acid; ND, not detected; PAST, pasteurized liquid infant formula; (72 °C x 10 s) frozen at −20 °C before use; SUHT, UHT‐treated formula stored at 40 °C for 60 days; UHT, indirect UHT‐treated liquid infant formula; (143 °C × 6 s) frozen at −20 °C before use.

### 2.3 Impaired Antibacterial Activity of IF

The bacterial growth curves for *Enterococcus faecalis* (10^4^ CFU mL^−1^) and *Staphylococcus epidermidis* (10^5^ CFU mL^−1^) were lower, and antibacterial activity thereby higher, when incubated with PAST than SUHT (*p* < 0.01), with UHT in between. The antibacterial activity in PAST was evident already at 2 h of incubation. At higher inoculation doses, differences appeared only at 6 h. For *E. faecalis* (10^5^ CFU mL^−1^), the inhibitory capacity of PAST was 28% higher than UHT (*p* < 0.05) and 62% higher than SUHT (*p* < 0.001), and for *S. epidermidis* (10^6^ CFU mL^−1^) the inhibitory capacity of SUHT was ≈60% lower than both PAST and UHT (*p* < 0.05). This indicates that SUHT had lower antibacterial activity compared to PAST and UHT.

### 2.4 Induction of NEC and Gut Dysfunction in Pigs Fed SUHT

**Figure 2:**
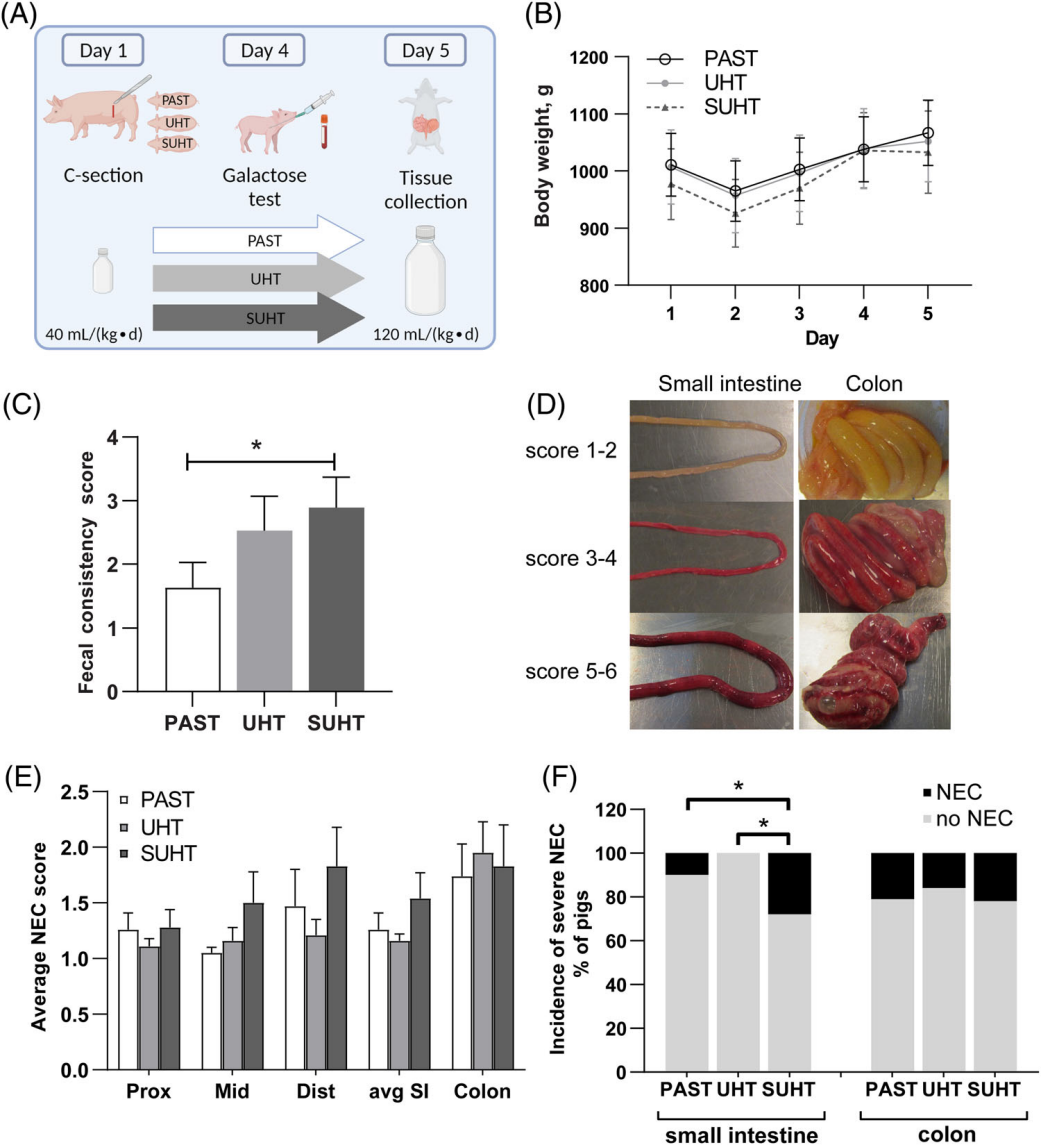
Clinical outcomes in preterm pigs fed PAST, UHT, and SUHT formula for 5 days. In vivo study design, outline created in Biorender.com A), body weight gain B), and diarrhea severity measured by fecal consistency score C). Representative images of NEC scoring system D), NEC scores E), and severe NEC incidence F) in the small intestine and colon. Values are mean ± SE. *p < 0.05. PAST, pasteurized liquid infant formula (72 °C, 10 s); SUHT, UHT‐treated formula stored at 40 °C for 60 days; UHT, indirect UHT treated liquid infant formula (143 °C, 6 s).

All pigs were fed for 5 days (**Figure** **2**A), except one pig fed SUHT that was euthanized on day 4 due to clear clinical signs of NEC. There were no differences in body weight (Figure 2B) or blood urea levels (an indicator of protein intake and metabolism, Table S2, Supporting Information). The pigs fed SUHT had more diarrhea (higher fecal consistency score) than pigs fed PAST (*p* < 0.05, Figure 2C). At euthanasia, small intestinal weight and weight‐to‐length ratio as an indicator of intestinal density were slightly lower in pigs fed SUHT than PAST, with intermediate values in pigs fed UHT (all *p* < 0.08, PAST vs pooled values of pigs fed UHT and SUHT, *p* < 0.05, Table S2, Supporting Information). The overall severity and incidence of NEC were relatively low across the small intestine and colon (NEC incidence 25%, average score <2), with lesions mostly located in the ileum and colon (Figure 2D,E). The incidence of severe NEC in the small intestine were though higher in pigs fed SUHT, compared with UHT and PAST (*p* < 0.05, Figure 2F).

**Figure 3:**
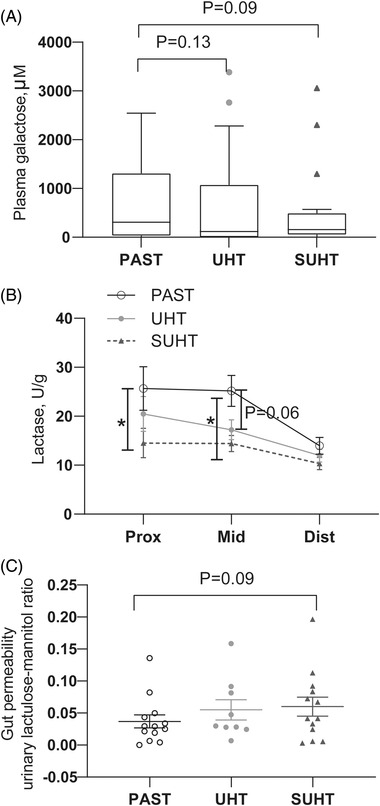
Intestinal function in pigs fed PAST, UHT, and SUHT formula for 5 days. In vivo galactose/glucose absorption on day 4 measured by plasma galactose concentration 20 min after oral ingestion of a bolus of galactose A). Intestinal digestive enzyme activities in proximal, middle, and distal small intestine B). Gut permeability represented by levels of non‐digestible and non‐absorptive lactulose passing through intestinal barrier and excreted into urine C). Values are mean ± SE. *p < 0.05. PAST, pasteurized liquid infant formula (72 °C, 10 s); SUHT, UHT‐treated formula stored at 40 °C for 60 days; UHT, indirect UHT‐treated liquid infant formula (143 °C, 6 s).

Intestinal functional analysis showed that pigs fed PAST tended to have higher galactose absorption than pigs fed SUHT and UHT (*p* = 0.09 and *p* = 0.13, respectively, **Figure** **3**A). Similarly, brush‐border lactase activity in the proximal and middle small intestine (both *p* < 0.05, Figure 3B) and dipeptidyl peptidase IV activity in the proximal small intestine (*p* = 0.1, data not shown) was higher in PAST than SUHT pigs, whereas aminopeptidase N activity was lower in pigs fed PAST than SUHT in the distal small intestine (*p* < 0.05, data not shown). No differences were found in the other brush‐border digestive enzyme activities measured, and sucrase and maltase activities were very low in all groups. Gut permeability measured by the lactulose–mannitol ratio tended to be higher in pigs fed SUHT versus PAST, with intermediate values in pigs fed UHT (*p* = 0.09, *p* = 0.19, respectively, Figure 3C).

### 2.5 Accumulation of MRPs and Amino Acid Cross‐Links in the Gut

**Figure 4:**
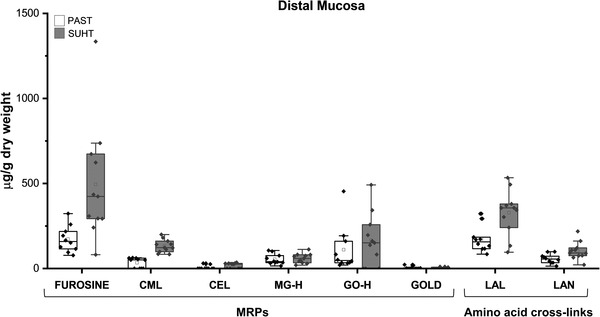
Maillard reaction products and cross‐links in ileal mucosa in preterm pigs fed PAST and SUHT formula for 5 days. Values are mean ± SE. *p < 0.05; **p < 0.01; ***p < 0.001. PAST, pasteurized liquid infant formula (72 °C, 10 s); SUHT, UHT‐treated formula (143 °C, 6 s) stored at 40 °C for 60 days.

Accumulation of MRPs and cross‐links in the ileal mucosa were investigated in pigs fed SUHT, the most physiologically affected group, and compared with PAST. Considerable amounts of furosine, CML, MG‐H, glyoxal derived hydroimidazolone (GO‐H), LAL, and LAN were detected in all pigs, whereas GOLD and CEL concentrations were mostly below the limit of detection. MOLD was not detected in any pigs. In general, all the compounds were detected in higher concentrations in pigs fed SUHT than PAST (**Figure** **4**), except for MG‐H and GO‐H, where the variations within the groups were higher. Furosine was the most abundant MRP measured and CML had the highest fold change (*p* < 0.001) in pigs fed SUHT compared with PAST, which is in accordance with the MRP levels in the IFs. Ileal levels of LAL and LAN were 2.3‐ and 1.6‐fold higher in pigs fed SUHT compared with PAST, respectively.

### 2.6 Inflammatory Response and Mucosal Barrier in Pigs Fed SUHT

**Figure 5:**
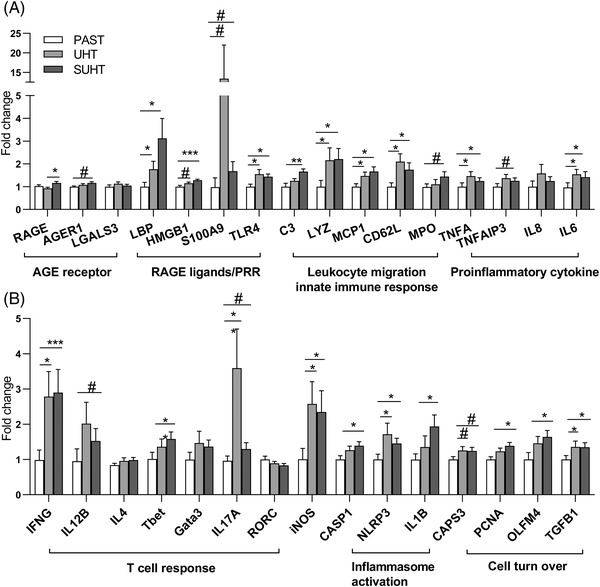
Gene expression in ileal mucosa of pigs fed PAST, UHT, and SUHT formula for 5 days. Intestinal inflammation and genes related to AGE–RAGE interaction A). Genes related to adaptive immune Th1 polarization, activation of inflammasome, oxidative stress, and cell turn over B). Data were normalized to the average expression level in the PAST group. PAST, pasteurized liquid infant formula (72 °C, 10 s); SUHT, UHT‐treated formula stored at 40 °C for 60 days; UHT, indirect UHT‐treated liquid infant formula (143 °C, 6 s).

Inflammatory immune mediators were measured in the ileal mucosa, where dietary MRPs predominantly accumulate.^[^ ^15^ ^]^ The expression of AGEs receptors (*RAGE* and *AGER1*) were slightly upregulated in pigs fed SUHT relative to the other groups with no changes in *LGALS3* (*p* < 0.05, *p* < 0.1, respectively, **Figure** **5**A). Ligands for RAGE and other pathogen recognition receptors (*LBP*, *TLR4*, *HMGB1*, *S100A9*) and most of the genes involved in acute inflammatory responses (*C3*, *TNFA*, *TNFAIP3*, *IL6*), leukocyte migration (*MCP1*, *CD62L*), and neutrophil bactericidal action (*LYZ*, *MPO*) were upregulated in pigs fed both UHT and SUHT, relative to PAST. Genes related to adaptive immune Th1 polarization (*IFNG*, *IL12B*, *TNFA*, *TNFAIP3*, *Tbet*), activation of inflammasome (*CASP1*, *NLRP3*, *IL1B*), oxidative stress (*iNOS*), and cell turn over (*CASP3*, *PCNA*, *OLFM4*, *TGFB1*) were upregulated primarily in pigs fed SUHT compared with PAST (*p* < 0.05), with intermediate values in pigs fed UHT (Figure 5B). Genes related to Th2 differentiation (*IL4*, *GATA3*) were not affected, whereas the Th17 cytokine (*IL17A*) was upregulated in both UHT‐fed groups with no changes in its transcription factor, *RORC*.

**Figure 6:**
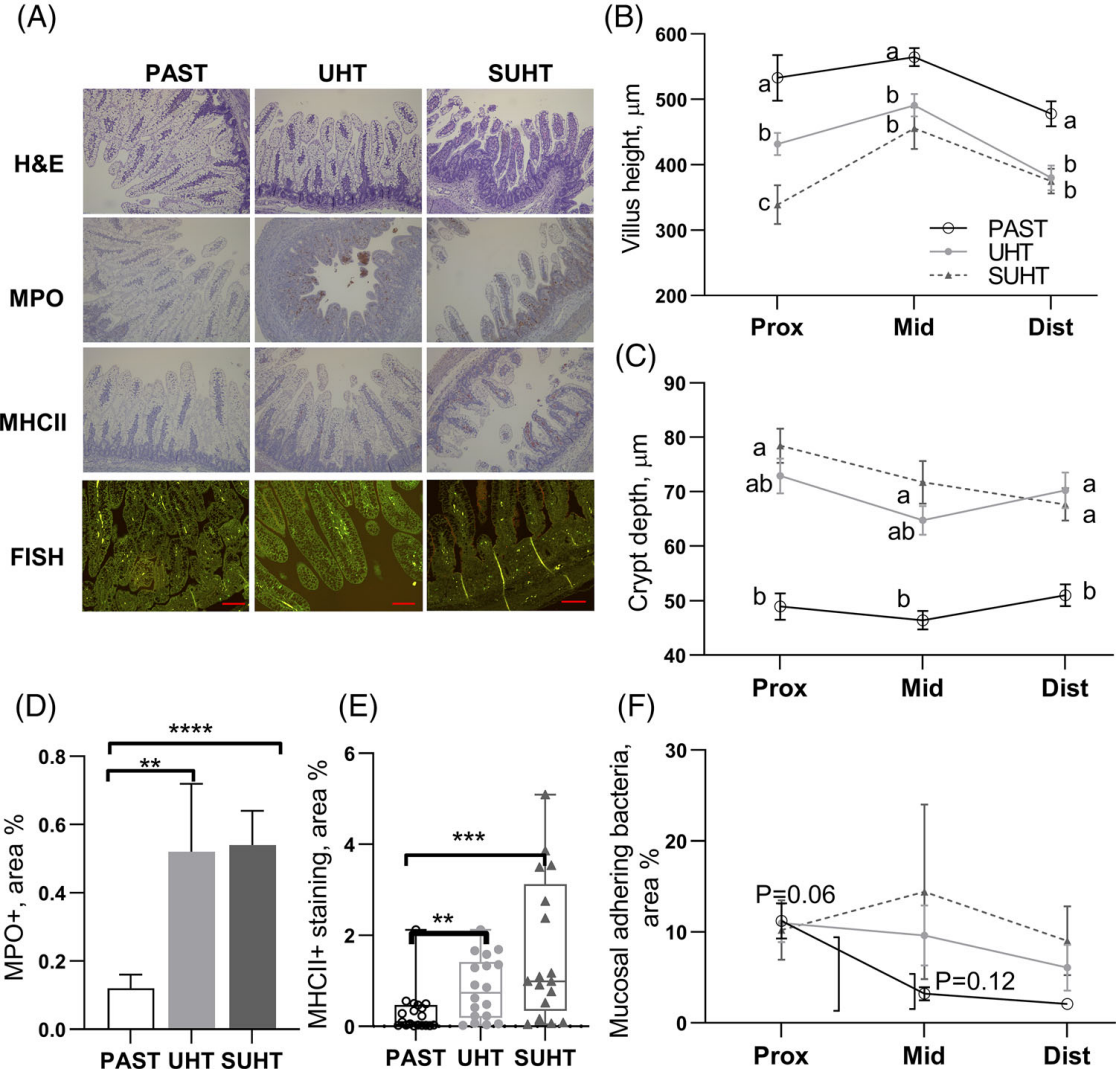
Intestinal morphology and mucosal adhering bacteria in pigs fed PAST, UHT, and SUHT formula for 5 days. Histological pictures A), villus height B), crypt depth C), density of MPO D), MHCII‐positive cells E), and density of mucosal adhering bacteria F). Values are mean ± SE. *p < 0.05; #, *p* < 0.1. PAST, pasteurized liquid infant formula (72 °C, 10 s); SUHT, UHT‐treated formula stored at 40 °C for 60 days; UHT, indirect UHT‐treated liquid infant formula (143 °C, 6 s).

Intestinal villus atrophy, loss of surface epithelium, and hyperplasia were also observed in pigs fed UHT and SUHT (with most severe pathology in SUHT‐fed pigs). This was reflected by reduced villus length and elongated crypt depth across the small intestine (*p* < 0.05, **Figure** **6**A–C). Further, in line with the higher myeloperoxidase (*MPO)* gene expression, there was an increase in MPO‐positive cells (i.e., monocytes and neutrophils) in pigs fed UHT and SUHT relative to PAST (*p* < 0.05, Figure 6D), and staining of MHCII as a marker of antigen‐presenting cells was increased in pigs fed UHT and SUHT (Figure 6E). The bacterial adhesion to the gut epithelium tended to be higher in pigs fed SUHT compared with PAST in the middle and distal intestine (*p* = 0.06 and 0.012, respectively, Figure 6F). Overall, SUHT‐fed pigs developed a more pronounced inflammatory response and gut structural damage than pigs fed UHT.

## 3 Discussion

We investigated how UHT treatment and storage of IF affected the immature gut in preterm pigs during 5 days of feeding as a clinically relevant model of preterm newborn infants in the immediate neonatal period. In this period, the immature gut is particularly sensitive to IF formulation.^[^ ^23^, ^24^ ^]^ With the emerging health concerns of MRPs, and especially AGEs, for infants^[^ ^12^, ^27^, ^28^ ^]^ and the increasing use of ready‐to‐feed liquid IFs for preterm infants in the neonatal intensive care units worldwide, this question remains crucial. Most studies investigating MRP effects have used extreme artificial heating conditions to facilitate Maillard reaction.^[^ ^14^, ^29^ ^]^ To characterize the impact of a clinically relevant ready‐to‐feed liquid IF in sensitive newborns, we applied indirect UHT treatment to an experimental liquid IF, and the stored UHT‐treated IF (40 °C for 60 days) represented a highly protein‐modified product derived from sub‐optimal storage. We showed that UHT treatment caused formation of large protein aggregates and MRPs accompanied by marked reductions in native bioactive proteins compared with a pasteurized IF (PAST) (Figure 1, Table 1).^[^ ^4^, ^5^, ^7^ ^]^ The extent of protein modifications were further exacerbated by subsequent storage at elevated temperature (SUHT) with significantly reduced antibacterial activity. Feeding UHT induced subclinical changes in the immature gut of the preterm pigs such as impaired lactase activity, activated innate immune responses and stunted villi, and feeding SUHT further caused accumulation of MRPs and an increase in expression of AGEs receptor in the intestine accompanied by clinical symptoms with more diarrhea, more severe NEC, a more pronounced inflammatory response, and functional and morphological damages relative to PAST (Figure 2). These adverse effects may be ascribed to a MRP‐mediated inflammatory response, oxidative stress, and lacking antimicrobial components in the UHT‐treated IFs.

The formation of non‐reducible protein cross‐links following UHT treatment and storage was confirmed by an increased concentration of LAL and LAN in UHT and SUHT (Table 1). LAL is known to be present in considerable concentrations in powdered IF (≈150–2100 µg g^−1^ protein) with even higher concentrations in ready‐to‐feed liquid IFs.^[^ ^30^, ^31^ ^]^ The formation of inter‐ or intra‐molecular LAL and LAN results in decreased digestibility and bioavailability of amino acids.^[^ ^32^ ^]^ This is in agreement with our previous study demonstrating lowered in vitro digestibility of the non‐reducible protein aggregates formed in SUHT.^[^ ^5^ ^]^ Moreover, LAL has been shown to be toxic to the kidneys of rats and mice.^[^ ^31^ ^]^ Although there is no certain knowledge about the toxicity of LAL in humans, it is recommended that its concentration is kept below 200 µg g^−1^ protein in IFs.^[^ ^31^ ^]^ In our study, the storage of IF markedly increased LAL and LAN concentrations, and the levels may be of concern particular for preterm infants with immature organs and deficient innate immune defence mechanisms.^[^ ^33^ ^]^

Concentrations of furosine, CML, CEL, MG‐H, and GO‐H in the IFs were increased by UHT treatment and further increased during storage (Table 1). As an acid derivative of the Amadori products formed from lactose, glucose, and galactose in IF during reactions with lysine residues after UHT treatment, furosine is a marker of early glycation.^[^ ^34^ ^]^ The furosine concentrations found in the present study are in agreement with the previously reported values across studies (214–19370 µg g^−1^ protein).^[^ ^35^ ^]^ With the progress of the Maillard reaction during UHT processing and storage, Amadori compounds degrade to form α‐dicarbonyl compounds. In the current study, UHT contained increased concentrations of the α‐dicarbonyl compounds glyoxal, methylglyoxal, glucosone, galactosone, 3‐deoxyglucosone, and 3‐deoxygalactosone (Table 1). These compounds react with lysine and arginine residues in proteins and lead to the formation of AGEs over time. This was confirmed by decreased α‐dicarbonyl compounds and following increase in CML, CEL, MG‐H, and GO‐H in SUHT. CML values in IF samples used in this study are comparable to previous findings for liquid IF (5.0–508 µg g^−1^ protein).^[^ ^35^ ^]^ Concomitantly, available lysine and arginine concentrations decreased by 10% in SUHT, possibly due to their modifications by carbohydrates and α‐dicarbonyl compounds during storage.

Feeding UHT‐treated IF in the first week of life induced inflammation and oxidative stress in the immature gut (Figure 5). The affected genes and proteins relate to the early inflammatory processes. This includes pathogen recognition via TLR4 signaling and Th17 differentiation (TLR4, LBP, IL17), early response Th1 cytokine expression (IL6, TNFA, IFNG), oxidative stress (iNOS), and innate immune activation mainly via neutrophil and monocyte function (S100A9, MPO, LYZ, MCP1).^[^ ^36^ ^]^ These acute inflammatory responses might explain the upregulation of intestinal apoptosis markers (CASP3), the villus atrophy, and the decreased gut function (Figure 5). Yet, these effects are considered mild and there was no sign of disruption in the gut epithelial layers given the minor changes in gut permeability and density of mucosal adhering bacteria. The subclinical gut dysfunctions might be partly explained by the diminished levels of native proteins and antimicrobial activities in UHT‐treated IF. After birth, newborn infants are rapidly colonized with microbes while the immune response, which is required to combat bacterial infections, is not yet fully functional.^[^ ^37^ ^]^ Therefore, the milk derived immune‐modulatory proteins become key antimicrobial and anti‐inflammatory components.^[^ ^38^ ^]^ Many of these bioactive proteins are heat‐sensitive,^[^ ^39^ ^]^ and the UHT treatment did cause losses of LF, IgG and IGF‐1 and antimicrobial activity (Figure 1), which are important for neutralizing bacteria and attenuating inflammatory responses, apoptosis and oxidative stress.^[^ ^40^ ^]^ This is consistent with our previous study, where increased heat load compromised the inhibitory effects of milk proteins on pathogen growth.^[^ ^41^ ^]^ The reduction in immune‐reactivity is most likely closely connected to the pronounced structural changes of the proteins, involving formation of disulfide bonds, an increase in the size of protein aggregates and formation of LAL and LAN as also observed previously.^[^ ^4^ ^]^

Detrimental effects on the immature gut became more obvious when pigs were fed SUHT as compared to UHT and PAST (Figures 2 and 3). This was accompanied by accumulation of AGEs in the ileum (Figure 4). The accumulated AGEs in tissues are most likely derived primarily from the IFs. While endogenous formation of AGEs in newborns is not well studied, accumulated data indicate that dietary AGEs represent a major contributor to the pool of AGEs in the body.^[^ ^42^ ^]^ This is supported by the findings that naturally suckled newborn pigs, which have almost no exposure to dietary AGEs, had no CML detected in their kidneys,^[^ ^29^ ^]^ whereas animals fed CML accumulated this component in all main organs with the highest abundance in the ileum.^[^ ^15^ ^]^ Direct effects of AGEs in promoting inflammation, proliferation and cell survival signaling have been observed in vitro.^[^ ^43^ ^]^ In our study, pigs fed SUHT were exposed to more dietary AGEs than pigs fed PAST and developed higher expression levels of genes related to gut mucosal inflammatory responses and Th1 immune activation as described above.

The composition of the experimental IFs was adapted to the nutritional value of IFs produced for infants for maximal translatability. Since normal newborn pigs have significantly higher needs for protein than infants (6 g 100 mL^−1^ porcine milk vs 1.5 g 100 mL^−1^ IF),^[^ ^44^ ^]^ the piglets received relatively low doses of protein in the current study. This is confirmed by the relatively low blood urea nitrogen levels, a marker of in vivo protein load and amino acid breakdown, observed in the pigs compared to many of our previous studies, where pigs were fed a higher protein content to meet their nutritional requirement. The damaging effects in the intestine were largest in response to SUHT, where more AGEs and non‐reducible aggregates were present, indicating a dose–response effect. It is unknown if these damaging effects would have been even more pronounced if an increased protein content in the IF was applied to fulfill the protein needs of the piglets, but we would hereby also risk to over‐interpret the results when translating to infants.

The amounts of aggregates and furosine in PAST were lower than in UHT, but was not free of these compounds. Pasteurization is considered a relatively mild heat treatment with limited impact on the Maillard reaction,^[^ ^45^ ^]^ and the modifications present in PAST likely originate from heat processing of the powdered whey protein ingredient.^[^ ^4^, ^7^ ^]^ Together with the reduced levels of native bioactive proteins, this may explain the NEC‐like lesions in the colon observed also in pigs fed PAST (Figure 2). The colon is more sensitive to NEC than the small intestine in newborn pigs, and we have previously documented improved gut structure and absorptive function and decreased permeability and NEC severity when feeding IFs compared with raw bovine milk, likely without MRPs, in preterm pigs.^[^ ^46^ ^]^

In conclusion, the current study characterized for the first time the effects of UHT treatment and storage of ready‐to‐feed liquid IFs on the immature gut of preterm neonates using a clinically relevant preterm pig model. An experimental IF subjected to indirect UHT treatment reduced protein bioactivity, which may contribute to the gut dysfunction and inflammation observed in the preterm pigs. Storage at elevated temperature accelerated the Maillard reaction, leading to AGEs accumulation and inflammation in the neonatal gut. Identifying the changes in protein modifications and MRPs in powdered and ready‐to‐feed liquid IFs from introduction to the market until end of shelf‐life would help to clarify the influence of processing and storage. Whether the adverse effects persist long‐term remains to be investigated, and the clinical consequences of using powdered versus liquid IFs for preterm newborn infants when human milk is not available should be further clarified.

## 4 Experimental Section

### Preparation of the Experimental IFs

The experimental liquid IFs were produced at Arla Foods Ingredients (Viby J, Denmark) as a complete generic liquid bovine‐based IF as described previously.^[^ ^4^ ^]^ The IFs were composed of 71% water, 16% skimmed milk, 4.7% lactose, 3.5% vegetable oil blend, 1.7% whey protein concentrate, and 0.04% lecithin. The remaining 3% consisted of a proprietary blend of vitamins, minerals, and carbohydrates including galactooligosaccharides and fructooligosaccharides. This resulted in a generic composition of 6.5% carbohydrates (of which more than 95% was lactose), 3.5% fat, and 1.5% protein (of which more than 60% was whey proteins), which mimics the nutrient composition in human milk. After blending, the IFs were homogenized (180 bar) and pasteurized at 72 °C for 10 s prior to use as control formula (PAST) or further subjected to UHT treatment by indirect tubular heating (143 °C, 6 s, SPX Flow technology, pilot plant type SPP, 100 L h^−1^, Søborg, Denmark) followed by cooling and homogenization (80 °C, 180 bar, SPX Flow, type RS‐14:38) and aseptic packing at 21 °C in clear polyethylene terephthalate bottles (500 mL, MONT11002, Grathwol, Karlslunde, Denmark) (UHT). The stored UHT‐treated liquid IF (SUHT) was obtained by storage in the dark at 40 °C for 60 days in an incubation cabinet (Binder, Tuttlingen, Germany). This storage condition was selected to mimic extreme temperature conditions observed during overseas transportation and in countries with high ambient temperatures.^[^ ^10^ ^]^ All prepared experimental IFs were immediately frozen at −60 °C until use. The experimental IFs used in this study were subjected to physicochemical characterization and in vitro digestion, and these results were discussed in detail in previous publications as part of a larger selection of experimental IFs.^[^ ^4^, ^5^, ^7^ ^]^ Selected results were included in the present paper in order to discuss the effects of processing and the physicochemical characteristics of the IFs on piglet gut health. Previously published results were indicated with references in Figures and Tables.

### Physicochemical Characteristics of IFs

CIELab color values of the IFs were measured in triplicates at three different positions on a petri dish covered with plastic wrap by a spectro2guide spectrophotometer (BYK‐Gardner GmbH, Geretsried, Germany) prior to freezing of IF samples. The total color difference (ΔE*) for UHT and SUHT was calculated relative to PAST in order to differentiate color difference caused by UHT treatment and storage as described previously.^[^ ^4^ ^]^ Further, pH was measured in triplicates in aliquots of the frozen (−60 °C) IF samples that were thawed in a water bath (25 °C, 1 h).

SDS‐PAGE was performed under reducing and non‐reducing conditions to evaluate disulfide‐linked aggregates and non‐reducible cross‐links in IFs.^[^ ^5^ ^]^ For reduction, 0.5 M DTT (Sigma‐Aldrich, St. Louis, MO, USA) was added to the samples to a final concentration of 50 mM DTT. Samples were standardized to the same protein concentration by dilution with Milli‐Q water to 1% w/v before addition of 4× NuPAGE LDS Sample Buffer (Thermo Fisher Scientific, Roskilde, Denmark) and heat treatment at 80 °C for 10 min. A total of 15 µg protein per well were loaded for each sample in triplicates of a precast 12% BisTris gel (NuPAGE, Thermo Fisher Scientific). Electrophoresis was run at 150 V for 90 min (Bio‐Rad, Hercules, CA, USA), and gels were stained with Coomassie Brilliant Blue R‐250 (Sigma‐Aldrich). The gels were destained in Milli‐Q water and scanned using an Epson Perfection V750 Pro scanner (Seiko Epson Corporation, Nagano, Japan). Comparison between gel bands was based on volumes computed by using Phoretix TL120 software, with a rolling‐ball 90 algorithm for background subtraction.

For Western blot analysis, samples were subjected to SDS‐PAGE under non‐reducing conditions (however, reducing conditions were applied for pro insulin‐like growth factor (IGF)‐1 due to its low immunogenicity) and transferred to a polyvinylidene difluoride membrane using the Iblot 2 system (Thermo Fisher Scientific). The membrane was then rinsed in 25 mL Tris‐buffered saline for a few seconds, followed by blocking of the membrane with 25 mL Tris‐buffered saline with 0.1% Tween 20 and 1% BSA (all Sigma‐Aldrich) for 4 h. After blocking, the membrane was incubated with the primary antibodies diluted in the blocking buffer, including pro IGF‐1 (1:2000, Thermo Fisher Scientific), bovine LF (1:30 000, Bethyl Laboratories, Montgomery, USA), and bovine IgG (1:3000, Thermo Fisher Scientific) overnight at 4 °C. The membrane was then washed in 25–30 mL Tris‐buffered saline with 0.1% Tween 20 for 6 × 5 min at room temperature on a rocking table. For IGF‐1 and LF, after the washing steps, the membrane was further incubated with secondary antibody (donkey anti‐goat IgG, horseradish peroxidase conjugated, 1:3000 dilution, Thermo Fisher Scientific) for 1 h at room temperature. The immunoreactive signals were developed using ECL reagent (Bio‐rad) and visualized by ChemiDoc Touch Imaging System (Bio‐rad). The level of bovine LF in the IFs was determined by ELISA (Bethyl Laboratories).

### Maillard Reaction Products and Amino Acid Cross‐Links

For quantification of MRPs and amino acid cross‐links in the IFs, AGEs, LAL, and LAN were quantified using the validated method described earlier.^[^ ^47^ ^]^ Briefly, IFs containing 3–5 mg protein were subjected to microwave‐assisted hydrolysis by using 6 M HCl (Honeywell Fluka, Fisher Scientific). Hydrolysates (500 µL) were evaporated to dryness in a vacuum concentrator (Savant SPD131DDA SpeedVac Concentrator; Thermo Fisher Scientific Inc., Waltham, MA, USA). The residues were dissolved in equal volume of Milli‐Q water and filtered through 0.22 µm regenerated cellulose syringe filters (Phenomenex, UK). Dilutions and addition of internal standards (Iris‐Biotech, Germany) were prepared so that the final sample solvent was acetonitrile:water (50:50, v/v). Five microliter sample was injected into the Dionex UltiMate 3000 LC system (Thermo Fisher Scientific) equipped with a Syncronis HILIC column (100 × 2.1 mm, 1.7 µm, Thermo Fischer Scientific) coupled to an OrbiTrap Q Exactive mass spectrometer (Thermo Fisher Scientific). The chromatographic conditions and mass spectrometric details were previously described.^[^ ^47^ ^]^ Quantification of furosine, CEL, MG‐H, GO‐H, LAL, LAN, lysine and arginine was performed based on internal standard calibration method by using stable isotopically labeled internal standards (Iris‐Biotech, Marktredwitz, Germany). MG‐H was expressed as MG‐H3 equivalents and GO‐H as GO‐H1 equivalents, as discussed previously.^[^ ^47^ ^]^ Analysis of CML in IFs was performed as previously described^[^ ^48^ ^]^ with minor modifications.^[^ ^7^ ^]^ Briefly, samples were reduced by using sodium borohydride (Sigma‐Aldrich) and hydrolyzed as described above. After evaporation of the acid and dissolving the residue in Milli‐Q water, 50 µL of CML‐d4 (400 ng mL^−1^) was added and the mixture was passed through OASIS Prime HLB cartridges (Waters, Denmark) and the eluent was collected. Sample (10 µL) was injected into an Acquity UPLC HSS T3 column (2.1 × 100 mm, 1.7 µm), which was equipped on the same LC‐MS system as described above. The method details were given elsewhere.^[^ ^7^ ^]^ α‐Dicarbonyl compound analysis was performed as previously described^[^ ^6^, ^49^ ^]^ with minor modifications.^[^ ^7^ ^].^ The IF (800 µL) was mixed with 1000 µL of ice‐cold methanol by using a vortex mixer and the mixture was incubated at −20 °C for 1 h. The contents were then centrifuged at 15,000 × *g* for 15 min at 4 °C. The supernatant (500 µL) was mixed with 150 µL phosphate buffer (0.5 M, pH 7.0) and 150 µL o‐phenylenediamine dihydrochloride solution (0.2%, w/v, Thermo Fischer Scientific) containing 18.5 mM diethylenetriaminepentaacetic acid (Sigma Aldrich). The contents were filtered immediately through 0.22 µm filters into UHPLC vials and incubated at 37 °C for 2 h in the dark for derivatization of the α‐dicarbonyl compounds. The quinoxaline derivatives of glucosone, deoxyglucosone, 3‐deoxyglucosone, 3‐deoxygalactosone, glyoxal, methylglyoxal, dimethylglyoxal were determined by LC‐MS as described before.^[^ ^7^ ^]^ Working solutions of glyoxal, methylglyoxal, dimethylglyoxal, and glucosone in the range of 10–200 ng mL^−1^ were derivatized and analyzed as described above to build the external calibration curve of quinoxaline, 2‐methylquinoxaline and 2,3‐dimethylquinoxaline, and glucosone quinoxaline forms, respectively. Quantification of 3‐deoxyglucosone was based on an external calibration curve prepared with 2‐(2′, 3′, 4′‐trihydroxybutyl)quinoxaline in Milli‐Q water within concentration range of 10–1000 ng mL^−1^. Galactosone and 3‐deoxygalactosone concentrations were quantified based on the standard curves of glucosone and 3‐deoxyglucosone, respectively, after confirming their identity with LC‐MS. For quantification of MRPs and amino acid cross‐links in intestinal tissues from pigs fed PAST and SUHT, freeze‐dried ileum mucosal tissues (containing 3–5 mg protein) were hydrolyzed in the same way as for IFs, and quantification was performed based on internal standard calibration as described above. For CML quantification, matrix‐match calibration was performed by using the hydrolysate of one sample, which was determined to contain no CML, as a blank. Calibration solutions of CML were prepared in the blank matrix at concentrations of 0–5 µg mL^−1^ and analyzed by LC‐MS/MS as described above.

### In Vitro Anti‐Bacterial Activity of Experimental IFs

The ability of the three IFs to inhibit the growth of *S. epidermidis* (WT1457, kindly provided by Xiaoyang Wang, University of Gothenburg, Sweden) and *E. faecalis* (isolated from a septic term infant, donated by Hvidovre Hospital, Denmark) were determined in vitro as previously described.^[^ ^41^ ^]^ Briefly, bacterial mid‐log culture stocks were prepared from single colonies of *S. epidermidis* and *E. faecalis*, grown overnight on 5% blood agar plates at 37 °C and subsequently inoculated in 10 mL of heart infusion broth. Following incubation overnight at the same condition, the OD of the bacterial cultures were adjusted to 0.05 at 600 nm (Ultrospec 2000, Pharmacia Biotech, Uppsala, Sweden) and continuously monitored until mid‐log phase was reached, upon which they were suspended in 15% glycerol and stored in aliquots at −80°C. For the antibacterial assay, the frozen bacterial culture stocks were thawed, centrifuged at 3200 × *g* and resuspended in sterile saline before incubation at 37 °C with the PAST, UHT, and SUHT at calculated doses of 10^6^ and 10^5^ CFU mL^−1^ for *S. epidermidis* and 10^5^ and 10^4^ CFU mL^−1^ for *E. faecalis*. The final inoculation doses and bacterial growth in the three liquid IFs after 2, 4 and 6 h of incubation were determined by counting CFUs after dilution plating on blood agar incubated overnight at 37 °C.^[^ ^41^ ^]^ Experiments were performed in triplicate and the bacterial inhibitory capacity of PAST compared to UHT and SUHT was calculated. Sterility of the three IFs were tested before inoculation. The PAST was not sterile; however, minor bacterial contamination was only apparent in one of the experimental replicates and the colony morphology was clearly distinguishable from the two bacterial strains tested.

### Animal Experiment

All animal procedures were approved by the Danish Animal Experiments Inspectorate (license number 2014‐15‐0201‐00418) and conducted in accordance with the EU directive 2010/63/EU for animal experiments. The ARRIVE guidelines 2.0^[^ ^50^ ^]^ was consulted for reporting of the animal experimental work. Sixty‐one preterm piglets (male:female ratio 25:36) were delivered from three sows by cesarean section at day 106 of gestation (Large White × Danish Landrace × Duroc; term = 116 ± 2 days), of which, five piglets were euthanized within 48 h due to respiratory stress and lung defects, thus 56 were included in the experimental groups. The pregnant sow was sedated with an intramuscular injection of a combination of zolazepam (25 mg mL^−1^) and tiletamin (25 mg mL^−1^) at a dose of 3 mL 100 kg^−1^ (Zoletil 50, Virbac, Kolding, Denmark), butorphanol at a dose of 2 mL 100 kg^−1^ (Dolorex, 10 mg mL^−1^, MSD Animal Health, Copenhagen, Denmark) and atropine at a dose of 2 mL (2care4, 1 mg mL^−1^, 2care4, Esbjerg, Denmark). Following sedation, a venflon was placed in an ear vein and universal anesthesia was induced with infusion of propofol (Propomitor, 10 mg mL^−1^, Orion Pharma Animal Health, Copenhagen, Denmark), until full anesthesia was achieved. The sow was then intubated and connected to a mechanical ventilator with inhalation of 2–5% isoflurane to maintain anesthesia (Isoflo Vet, Zoetis, Farum, Denmark). The sow was placed in lateral recumbency and the surgical site aseptically prepared before 50 mL of local infiltration anesthesia, i.e., lidokain (Lidor Vet, 20 mg mL^−1^, Salfarm, Kolding, Denmark) was applied. The flank was incised and the fetuses were delivered through incisions in the large uterine curvature. The umbilical cord of each piglet was ligated and transected, and blood from the sow was aseptically collected and used later for passive immunization of the piglets. The sow was subsequently euthanized with intravenous infusion of 100 mL pentobarbital sodium (Euthanimal, 400 mg mL^−1^, ScanVet Animal Health, Fredensborg, Denmark).^[^ ^51^ ^]^ Immediately after delivery and upon successful resuscitation including intramuscular administration of 0.1 mL doxapram (Dopram, 20 mg mL^−1^, Carinopharm GmbH, Eime, Germany) and 0.1 mL flumazenil (0.1 mg mL^−1^, Hameln pharma GmbH, Hameln, Germany) and if necessary positive‐pressure ventilation, piglets were transferred to the intensive care unit and placed in individual incubators with regulated temperature (37–38 °C) and oxygen supply (0.5–2.1 min^−1^, within the first 24 h). Utilizing the sow‐transferred anesthetics, each piglet was fitted with an indwelling vascular catheter (infant feeding tube, 4F; Portex, Kent, UK) inserted into one of the umbilical arteries with the tip of the catheter reaching the dorsal aorta for continuous parenteral nutrition and blood sampling. An orogastric feeding tube (6F Portex) was passed through the cheek and secured with sutures to facilitate enteral nutrition. Following catheterization, all piglets were immunized systemically with arterial administration of plasma isolated (4000 × *g*, 4 °C, 10 min) from their own mother at 4, 12, and 20 h after delivery at 4, 5, and 7 mL kg^−1^, respectively.^[^ ^51^ ^]^ Pigs were stratified into three groups (*n* = 18–19) according to body weight and sex, and were fed increasing volumes of PAST, UHT, or SUHT through the orogastric feeding tube every 3 h, from 40 to 64 mL (kg d)^−1^ on days 1–2 to 100–120 mL (kg d)^−1^ on days 3–5. During the study period, the pigs also received gradually decreasing doses of parental nutrition (Kabiven with added Vamin 18 g N/L, Vitalipid (10 mL), Tracel (10 mL), and Soluvit (10 mL), all Fresenius Kabi, Uppsala, Sweden, and 250 U L^−1^ heparin (Leo Pharma, Ballerup, Denmark)). Glucose and fat were withdrawn to reach a final nutrient concentration of total energy, 2899 kJ L^−1^; protein, 44 g L^−1^; glucose, 71 g L^−1^; and lipids, 31 g L^−1^. The parenteral nutrition was infused through the umbilical catheter using syringe infusion pumps (Infusomat Secura; Braun, Kronberg, Germany) (96–48 mL (kg∙d)^−1^) to ensure adequate nutrient and fluid intake.^[^ ^44^ ^]^

### Clinical Assessment and In Vivo Gut Function

Pigs were closely monitored by blinded personnel at least every 3 h, and in case of severe clinical complications (e.g., severe pain, low blood perfusion, respiratory distress) piglets were sedated with 0.1 mL kg^−1^ intramuscularly administered Zoletil mix (zolazepam and tiletamin (Zoletil 50, Virbac), xylazine (Xysol Vet, 20 mg mL^−1^, ScanVet Animal Health), ketamine (Ketaminol Vet, 100 mg mL^−1^, MSD Animal Health), and butorphanol (Dolorex, MSD Animal Health)) and finally euthanized with an intra‐cardiac injection of pentobarbital sodium (Euthanimal, ScanVet Animal Health). The same euthanasia protocol was used on day 5 prior to tissue collection. Fecal consistency was assessed twice daily using a scoring system ranging from 1 (firm feces) to 6 (severe diarrhea) with a score ≥3 indicating diarrhea. Gastric emptying was evaluated by measuring the proportion of stomach content at euthanasia relative to an oral feeding bolus given 2 h before euthanasia. Intestinal hexose absorptive capacity was determined by measuring the increment in plasma galactose 20 min after receiving an oral bolus of 15 mL kg^−1^ of a 10% galactose solution on day 4, as previously described.^[^ ^52^ ^]^ Briefly, for analysis of galactose, deproteinized samples of plasma were added to an NAD solution (Sigma‐Aldrich). Subsequently, galactose dehydrogenase (Sigma‐Aldrich) was added, catalyzing the reaction between plasma galactose and NAD. The amount of NADH formed from the reaction was detected spectrophotometrically at 340 nm (Pentra C400, Horiba Europe GmbHHH). Similarly, gut permeability was determined by measuring the urine lactulose–mannitol ratio in response to an oral bolus of 10 mL kg^−1^ solution containing 7.5% lactulose and 7.5% mannitol (both Sigma Aldrich) given 3 h before euthanasia. Concentrations of mannitol and lactulose in urine collected by puncture of the urinary bladder after euthanasia were analyzed as previously described.^[^ ^51^ ^]^

### Tissue NEC Evaluation and Function

The proximal (Prox), middle (Mid), and distal (Dist) small intestine and colon were dissected and macroscopically evaluated for NEC lesions using a scoring system ranging from 1 to 2 (absent of lesion or minimal local hyperemia), 3–4 (extensive hyperemia or hemorrhage), to 5–6 (transmural necrosis or pneumatosis).^[^ ^53^ ^]^ Animals with severe NEC were defined as pigs with a score ≥3 in at least two small intestinal regions or a score ≥4 in at least one region of the small intestine or colon. Small intestine and scraped ileal mucosa were snap‐frozen or fixed in 4% paraformaldehyde solution (Hounisen Laboratorieudstyr, Skanderborg, Denmark) for further analyses. Intestinal brush‐border enzymes for final digestion of luminal nutrient oligomers, including lactase, maltase, sucrase, aminopeptidase N, aminopeptidase A, and dipeptidyl peptidase IV, were analyzed in frozen small intestinal tissue. Activities were determined spectrophotometrically using lactose, sucrose, maltose, glycyl‐l‐prolin‐4‐nitroanilide, l‐alanine‐4‐nitroanilide, and a‐l‐glutamic acid 4‐nitroanilide respectively, as substrates, in accordance with a previously established protocol.^[^ ^54^ ^]^

Gene expression levels were determined in the ileal mucosa by quantitative PCR.^[^ ^55^ ^]^ Briefly, mRNA was extracted from frozen tissues using RNeasy Lipid Tissue Mini Kit (Qiagen, Hilden, Germany), and was converted to cDNA using High‐Capacity cDNA Reverse Transcription Kit (Cat. No. 4368814, Applied Biosystems). Primers to amplify target genes (TAG Copenhagen A/S, Copenhagen, Denmark) were listed in Table S1, Supporting Information. Expression levels of target genes were quantified using LightCycler 480 (Roche Life Science, Basel, Switzerland) and were normalized to hypoxanthine‐guanine phosphoribosyltransferase (HPRT1) as reference gene.

### Intestinal Mucosal Morphology and Bacterial Adhesion

Paraformaldehyde‐fixed and paraffin‐embedded small intestinal samples were stained with hematoxylin and eosin to characterize mucosal morphological changes as indicated by villus length and crypt depth. Immunohistochemical staining with anti‐MPO (Rabbit Anti‐Human, A0398, Dako, Agilent Technologies, Glostrup, Denmark) and anti‐MHCII antibodies (Mouse Anti‐Pig SLA CLASS II DQ, MCA1335GA, Bio‐Rad Laboratories, Copenhagen, Denmark) were performed on ileal sections (5 µm). Staining was developed with ABC (Avidin‐Biotin Complex) kits for MPO staining and UltraVision LP Detection System (Thermo Fisher Scientific) for MHCII staining using DAB and AEC as chromogens. All specimens were counterstained with Mayer's hematoxylin. Images were acquired using the Leica LAS EZ software (version 3.4.0, Leica Microsystems, Brønshøj, Denmark) and the proportion of positive staining was analyzed using the IHC toolbox in ImageJ (National Institutes of Health, Bethesda, MD, USA). Fluorescence in situ hybridization was performed on paraffin‐embedded small intestinal sections to quantify the amount of mucosal adhering bacteria, using an Alexa Fluor 555‐labeled universal DNA‐probe (nucleotide sequence: 5’‐GCTGCCTCCCGTAGGAGT‐3’) targeting 16S rRNA (Eurofins Genomics, Ebersberg, Germany) as previously described.^[^ ^56^ ^]^ Stained sections were digitalized using an Axioscan 7 slide scanner (Zeiss, Jena, Germany), and Alexa Fluor 555 signal and autofluorescent background tissue area segmented and quantified using ZEN Intellesis software (Zeiss).

### Statistical Analyses

All data were analyzed using the statistical software R version 3.5.0. Primary analyses were performed among PAST, UHT, and SUHT and the similar pig treatment groups. Sub‐analyses to determine overall effects of ultra‐high temperature treatment were performed by comparing the PAST treated pigs with pooled values of UHT and SUHT‐treated pigs. Continuous outcomes (e.g., organ weight, hematology, gene expression, etc.) were analyzed using a linear model (*lm* function) and a *p*‐value for, e.g., “treatment” was generated by F test. Binary outcomes such as NEC incidence was analyzed by binomial logistic regression using a generalized linear model (*glm* function). Ordinal outcomes (e.g., fecal consistency and NEC score) were analyzed using the cumulative link model (*clm* function, *ordinal* package). For binary and ordinal outcomes, a *p*‐value for “treatment effect” was tested by a likelihood ratio test. The above analyses were followed by Tukey test for pairwise group comparisons. Outcomes with repeated measurements (e.g., enzyme activities in more than one intestinal segment, daily body weight change) were analyzed by linear mixed‐effect models with pig as a random factor (*lmer* function). This was followed by post‐hoc group comparisons of treatment level mean at each measurement point using least‐squares means estimate and general linear hypothesis testing with *glht* function (*lsmeans* and *multcomp* package). All models were adjusted for potential confounders as covariates (i.e., litter, gender, birth weight).

## Conflict of Interest

C. F. Nielsen is employed at Arla Foods Ingredients. D. E. W. Chatterton, P. T. Sangild, M. N. Lund, and S. B. Bering have received funding from Arla Foods Ingredients. The other authors have no conflict of interest.

## Author Contributions

J.S. and H.G.A. are considered equal first authors of the work. M.N.L. and S.B.B. are considered equal senior authors of the work. J.S., H.G.A., D.E.W.C., P.T.S., M.N.L., S.B.B. conceptualized and designed the study. J.S., H.G.A., K.A.‐O., P.L., Y.Y., X.Z., A.B. performed the experiments and analyses. J.S., H.G.A., M.N.L., and S.B.B. wrote and edited the manuscript. C.F.N. contributed to conceptualization of the study. M.N.L. and D.E.W.C. were responsible for funding acquisition. All authors reviewed and approved the final version of the manuscript.

## Supporting information



Supporting Information

## Data Availability

The data that support the findings of this study are available from the corresponding author upon reasonable request.
