# Supplementary material for: Ultra‐High Temperature Treatment and Storage of Infant Formula Induces Dietary Protein Modifications, Gut Dysfunction, and Inflammation in Preterm Pigs
Source: Mol Nutr Food Res. 2022 Sep 12;66(20):2200132. doi: 10.1002/mnfr.202200132 (PMC9786312; doi:10.1002/mnfr.202200132)
Supplement: Supplementary file 1 — Supporting Information [file MNFR-66-2200132-s001.pdf]

Supplementary Table 1. Primer sequences used in real-time quantitative PCR analysis.

**Gene**	**Forward (5'-3')**	**Reverse (5'-3')**	**References**
RAGE	AGGTGGAGCTGTACCTCCTG	TCCTTGGTCCAGTGGATTTG	doi:10.1186/s12974-018-1201-x
AGER1	CAGCATCGTGATTGAGCAGC	CAGGCGGTTGTAGTCCACTT	
LGALS3	CGCTTCAACGAGGACAACAG	CGCAACCTTGAAGTGGTCAG	
LBP	CCCAAGGTCAATGATAAGTTGG	ATCTGGAGAACAGGGTCGTG	
HMGB1	AGGATCCCAATGCACCCAAG	TGCAGCGGTGTTATTCCACA	
S100A9	TGCCAATCAAGACGAACAGGT	ACTCCTTGTGGATGTTGTCGT	
TLR4	TGGTGTCCCAGCACTTCATA	CAACTTCTGCAGGACGATGA	doi:10.2217/epi-2017-0122
C3	GCTCCACGGTCCTCTATCG	TGCCAAACTGGTTGTGGGAT	doi:10.1002/jpen.1422
LYZ	TAAAGCATGGGTGGCATGGA	CAGTTTGCAACCCCGAATGT	
MCP1	ATCTGTGCAGAACCCAAGCA	TCAAGGCTTCGGAGTTTGGT	
CD62L	GGAGCAAAGACTCCGGGAAG	CATGACCTGGGCTGACAAGA	doi:10.1186/s12974-018-1201-x
MPO	GGTCCAGATCATCACTTACCG	GGTCCACCGAGTCATTGTAG	doi:10.1002/jpen.1422
TNFA	ATTCAGGGATGTGTGGCCTG	CCAGATGTCCCAGGTTGCAT	doi:10.1186/s12974-018-1201-x
TNFAIP3	CCCAGCTTTCTCTCATGGAC	TTGGTTCTTCTGCCGTCTCT	
IL8	CTGTGAGGCTGCAGTTCTGG	CCAGGCAGACCTCTTTTCCAT	doi:10.2217/epi-2017-0122
IL6	TGGGTTCAATCAGGAGACCT	CAGCCTCGACATTTCCCTTA	doi:10.1016/j.clnu.2013.05.013
IFNG	CGATCCTAAAGGACTATTTTAATGCAA	TTTTGTCACTCTCCTCTTTCCAAT	doi:10.1016/j.jim.2005.06.021
IL12B	TCTTGGGAGGGTCTGGTTTG	AAGCTGTTCACAAGCTCAAGTATGA	doi:10.4049/jimmunol.1600672
IL4	TCTCACCTCCCAACTGATCC	AGCTCCATGACGAGTTCTT	doi:10.1021/jf803133b
Tbet	CTGAGAGTCGCGCTCAACAA	ACCCGGCCACAGTAAATGAC	doi:10.3389/fimmu.2019.02402
GATA3	ACCCCTTATTAAGCCCAAGC	TCCAGAGAGTCGTCGTTGTG	doi:10.3389/fimmu.2019.02402
IL17A	ATCCCACAAAGTCCAGGATG	GTGTGCTCCGGTTCAAGAT	doi:10.1002/jpen.1422
RORC	CAGCGCTCCAACATCTTCTC	GACCAGCACCACTTCCATTG	doi:10.3389/fimmu.2019.02402
iNOS	CAACAATGGCAACATCAGG	CATCAGGCATCTGGTAGC	
CASP1	CTCTCCACAGGTTCACAATC	GAAGACGCAGGCTTAACTGG	doi:10.1080/08820139.2017.1360341
NLRP3	AGCAGATTCCAGTGCATCAAAG	CCTGGTGAAGCGTTTGTTGAG	doi:10.1080/08820139.2017.1360341
IL1B	TCTGCATGAGCTTTGTGCAAG	ACAGGGCAGACTCGAATTCAAC	

CAPS3	ATTGAGACGGACAGTGGGAC	GCTGCACAAAGTGACTGGAT	
PCNA	GCAGATGTACCCCTTGTTGT	AGTATGTGCTGGCATCACCG	doi:10.1002/jpen.1422
OLFM4	CGAATCCCAGTCGGTTTCCA	TGATTTCCAAGCGCTCCACT	
TGFB1	CGTGCTAATGGTGGAAAGCG	TCTGCCCGAGAGAGCAATAC	
HPRT1	TATGGACAGGACTGAACGGC	ATCCAGCAGGTCAGCAAAGA	doi:10.2217/epi-2017-0122

Supplementary Table 2. Organ weights and blood biochemistry in pigs fed PAST[a], UHT and SUHT formula for 5 days.

Parameters	PAST	UHT	SUHT
Organ weights			
Stomach, g/kg	5.9 ± 0.2[b]	5.8 ± 0.3	5.6 ± 0.2
Prox small intestine, g/kg	10.1 ± 0.4	9.7 ± 0.4	9.3 ± 0.4[#]
Mid small intestine, g/kg	12 ± 0.5	11.3 ± 0.4	11.8 ± 0.6
Dist small intestine, g/kg	11.4 ± 0.4	10.6 ± 0.4	10.6 ± 0.5[#]
Total small intestinal weight, g/kg	33.5 ± 1.0	31.6 ± 1.1	31.8 ± 1.4[#]
Small intestinal length, cm/kg	338 ± 15	332 ± 15	335 ± 14
Small intestinal weight-length-ratio, g/cm	0.102 ± 0.004	0.098 ± 0.005	0.097 ± 0.005[#]
Colon, g/kg	15.7 ± 1.3	15.0 ± 1.1	15.8 ± 1.2
Spleen, g/kg	1.83 ± 0.09	1.8 ± 0.08	1.98 ± 0.06
Heart, g/kg	7.5 ± 0.2	7.3 ± 0.3	6.7 ± 0.2*
Kidney, g/kg	8.6 ± 0.3	8.6 ± 0.3	8.0 ± 0.3
Liver, g/kg	31.7 ± 0.7	31.9 ± 1.3	31.6 ± 0.9
Lung, g/kg	24.7 ± 1.1	24 ± 1.1	23.8 ± 1.1
Cerebellum, g	2.49 ± 0.06	2.58 ± 0.05	2.51 ± 0.05
Cerebrum, g	22.0 ± 0.3	22.0 ± 0.4	21.9 ± 0.3
Hippocampus, g	0.44 ± 0.02	0.46 ± 0.01	0.45 ± 0.01
Striatum, g/kg	0.25 ± 0.01	0.27 ± 0.01	0.27 ± 0.01
Mid brain, g	2.6 ± 0.05	2.71 ± 0.05	2.64 ± 0.04
Brain water content, %	85.15 ± 0.06	85.32 ± 0.08	85.14 ± 0.11
Blood biochemistry			
Serum albumin, g/L	9.5 ± 0.3	9.7 ± 0.3	9.2 ± 0.3
Alkaline phosphatase, U/L	2463 ± 187	2086 ± 214	2088 ± 120
Alanine aminotransferase, U/L	16.3 ± 0.5	16.7 ± 0.5	17.6 ± 1.4
Aspartate aminotransferase, U/L	35.7 ± 5.9	37.1 ± 4.8	63 ± 21
Calcium, mmol/L	2.98 ± 0.06	2.95 ± 0.04	2.92 ± 0.04
Cholesterol, mmol/L	2.30 ± 0.09	2.13 ±0.08	2.2 ± 0.1
Creatinine kinase, U/L	136 ± 28	179 ± 33	305 ± 115
Creatinine, µmol/L	57 ± 2	58 ± 2	58 ± 2
γ -Glutamyltransferase, U/L	32.6 ± 2.0	32.5 ± 2.5	36.1 ± 3.6
Glucose, mmol/L	3.41 ± 0.23	3.63 ± 0.32	3.57 ± 0.29
Phosphate, mmol/L	1.48 ± 0.05	1.52 ± 0.05	1.63 ± 0.18
Iron, µmol/L	10.5 ± 1.6	8.9 ± 1	11.7 ± 1.1
Potassium, mmol/L	4.14 ± 0.09	4.23 ± 0.09	4.42 ± 0.29
Lactate, mmol/L	1.28 ± 0.11	1.68 ± 0.34	1.59 ± 0.38
Magnesium, mmol/L	0.82 ± 0.03	0.82 ± 0.03	0.82 ± 0.03
Sodium, mmol/L	147 ± 1	146 ± 1	145 ± 0
Total bilirubin, µmol/L	2.63 ± 0.26	2.74 ± 0.3	3.33 ± 0.57
Total protein, g/L	27.5 ± 0.7	27.7 ± 0.5	26.6 ± 0.5
Blood urea, mmol/L	0.66 ± 0.05	0.63 ± 0.05	0.90 ± 0.29

[a] PAST, pasteurized liquid infant formula (72 °C x 10 s); UHT, indirect UHT treated liquid infant formula (143 °C × 6 s); SUHT, UHT treated formula stored at 40 °C for 60 days.

[b] Values are mean ± SEM. *, $p<0.05$; #, $p<0.1$ when comparing to the PAST group.
